# Cross-sectional research into counselling for non-physician assisted suicide: who asks for it and what happens?

**DOI:** 10.1186/1472-6963-14-455

**Published:** 2014-10-02

**Authors:** Martijn Hagens, H Roeline W Pasman, Bregje D Onwuteaka-Philipsen

**Affiliations:** Department of Public and Occupational Health, Expertise Centre for Palliative Care, VU University Medical Centre, EMGO+ Institute for Health and Care Research, Van der Boechorststraat 7, 1091 BT Amsterdam, The Netherlands

**Keywords:** Counselling, Non-physician assisted suicide, Suicide/assisted suicide, Foundation De Einder, Suicide-attempt prevention, Self-determination, Autonomy

## Abstract

**Background:**

In the Netherlands, people with a wish to die can request physician assistance in dying. However, almost two thirds of the explicit requests do not result in physician assistance in dying. Some people with a wish to end life seek counselling outside the medical context to end their own life. The aim of this cross-sectional research was to obtain information about clients receiving counselling for non-physician assisted suicide, and the characteristics and outcome of the counselling itself.

**Methods:**

All counsellors working with foundation De Einder (an organisation that offers professional counselling for people with a wish to end life) (N=12) filled in registration forms about all clients they counselled in 2011 and/or 2012. Only client registration data forms with at least one face-to-face contact with the counsellor were selected for analysis (n=595).

**Results:**

More than half of the clients were over 65 years old. More than one third of the clients had no wish to end life and 16% had an urgent wish to end life. Almost two thirds of the clients had not requested physician assistance in dying. Half of the clients had others involved in the counselling. More than half of the clients received explicit practical information concerning non-physician assisted suicide, while 13% of all clients actually ended their own life through non-physician assisted suicide. Clients without a (severe) disease were older than clients with a severe disease. They also had more problems of old age and existential suffering and more often wanted to be prepared for self-determination. The clients without a (severe) disease more often had no wish to end life and requested physician assistance in dying less often than clients with a severe disease.

**Conclusion:**

While some of the clients receiving counselling for non-physician assisted suicide seem to be looking for a peaceful death to escape from current suffering, others have no wish to end life and seem to be looking for reassurance in anticipation of prospective suffering. If non-physician assisted suicide is be distinguished from ‘mutilating’ suicide, this asks for a different approach than suicide crisis intervention, for example suicide-attempt prevention.

**Electronic supplementary material:**

The online version of this article (doi:10.1186/1472-6963-14-455) contains supplementary material, which is available to authorized users.

## Background

Death wishes occur in about 10% of the general population in the Netherlands [1]. The ones acting upon this wish can roughly be divided into three categories: suicide, physician assisted dying (PAD) and non-physician assisted suicide (non-PAS).

In the Netherlands, people with a wish to die can request physician assistance in dying under the Termination of Life on Request and Assisted Suicide Review Procedures Act [2]. In 2010 2.9% of all annual deaths – about 4,000 people – occurred through PAD [3]. However, it is known that many patients that request physician assistance in dying do not receive it [3, 4]. PAD is only allowed when an underlying physical or psychiatric disease is present and the legal criteria of due care are met [2]. But even when it is permissible, physicians are reluctant to provide PAD for patients with dementia, psychiatric diseases and elderly people who are weary of life [5]. In the Netherlands, in 2010 about 13,400 patients explicitly requested physician assistance in dying, and another 33,900 requested physician assistance in dying for at an undetermined future time [5].

In the Netherlands, suicide accounts for 1.25% of all annual deaths (1,753 in 2012) [6, 7]. It is estimated there are an additional 14,000 to 16,000 non-fatal suicide attempts per year [8]. In about half of the suicides, mental disorders are the underlying motive. The majority of these suicides occur through a ‘mutilating’ method (like hanging, drowning or jumping), and therefore often occur in solitary and isolated circumstances [7].

In the past few years in the Netherlands, more attention has been given to ‘self-euthanasia’ or ‘non-physician assisted suicide’ (non-PAS). These suicides are characterized by a non-mutilating method, like voluntary refusing food and fluid, taking lethal medication or oxygen deprivation by inhalation of inert gas. These suicides are regarded to be more well-considered, more carefully prepared and more often with openness towards others than seen with ‘mutilating suicides in solitary and isolated circumstances’ [9–12]. Although a physician can be involved with non-PAS (for example by providing care during the process of voluntary refusing food and fluid), there is an important difference from PAD. With non-PAS a medical professional does not carry the responsibility for distributing or administering the means that cause death, as under the Dutch PAD law. It’s estimated that in 2010 between 0.4% and 2.1% of all annual deaths have occurred through voluntarily refusing food and flood and between 0.2% and 1.1% of all annual deaths through taking lethal medication [13, 14]. The prevalence of oxygen deprivation by inhalation of inert gas has not been researched in the Netherlands, but a rise in its occurrence has been noted in the Netherlands [15] and abroad [16].

While ‘mutilating’ suicides and PAD have been more often researched, less is known about people that want non-PAS. They have the option to look for information on and counselling for non-PAS as provided by – for example – foundation De Einder (see Table 1 for founding history, goal and work method of foundation De Einder). As little information is available on the trajectory of counselling non-PAS we focus on answering the following research questions:

- What are the characteristics and underlying sources of suffering of people receiving counselling for non-PAS?
- What are the characteristics and outcome of the counselling for non-PAS?
- Are there differences in the characteristics and underlying sources of suffering of people receiving counselling for non-PAS and characteristics and outcome of this counselling between people with a severe (or terminal) illness and people without a (severe) illness?

**Table 1:** **Foundation De Einder**

Topic	
Founding history	Foundation De Einder was founded in 1995 as a result of dissatisfaction with the situation that people with a wish to end life were “being left out in the cold”.
Goal	The goal of the foundation is “to promote and – if deemed necessary – to offer professional counselling for people with a wish to end life who ask for help, with respect for the autonomy of the person asking for help […]” [17].
	Contrary to suicide prevention or crisis intervention organisations, foundation De Einder regards suicide as a possible outcome and gives information about non-physician assisted suicide (non-PAS). Autonomy is regarded as an important value. Seen as an addition to the – since 2001 in the Netherlands legalized – medicalized approach of physician assistance in dying, foundation De Einder refers people who seek help to independent counsellors to offer counselling focused on non-PAS, which is a demedicalized approach.
Work method	The work of these counsellors entails non-directive counselling and consists of having conversations, offering mental support and providing general information on non-physician assisted suicide. These three forms of assistance by lay persons are regarded as legal assistance in suicide [18].
	The counselling is aimed at creating an as large as possible clarity regarding the wish to end one’s life and possible suicide. This covers the mental process of decision-making and might include matters like considering alternatives, timing of death, and consideration of others. In the situation the client decides to act upon the wish to end life, the counselling is aimed at realising the best possible preparations for non-PAS. This covers the practical preparation and might include gathering means for and the effectuation of the suicide [12, 17].
	The counselling is not aimed at a certain choice or direction, but is aimed at attaining the highest possible quality of the choice and – if it comes to that – the highest possible quality of implementation of the wish to end life [17].

## Methods

### Design

Data was collected from annual registration forms that counsellors working together with foundation De Einder filled out for all clients they had contact with that year.

### Population

Data collection over the years 2011 and 2012 took place in the first two months of the consecutive year. All counsellors working in cooperation with De Einder in 2011 and 2012 (N = 12) filled out the registration form (response rate 100%). Eight of them filled in the data for two years, while two only for 2011 and two others only for 2012. This resulted in data of 547 clients from 2011 and 444 clients from 2012. Only clients with whom at least one face-to-face contact had occurred with the counsellor were included in this study, because clients without face-to-face contact are usually in an orientating phase where counselling consists of offering general information and/or moral support. Furthermore counsellors only share explicit information about non-PAS (concerning gathering the means for and the effectuation of the suicide) during face-to-face contacts due to the sensitivity of the information. This resulted in 325 clients in 2011 and 310 in 2012. Forty clients who were registered both years were excluded from the oldest dataset to ensure the most recent information about these clients was available. This resulted in a total number of 595 clients for analysis.

### Measurement instruments

The researcher digitalised the registration form that was previously used by the board of foundation De Einder and – in consultation with counsellors – expanded the form. To increase reliability and uniformity and to avoid bias, several meetings with the counsellors were held to explain the instructions for filling out the form. After counsellors filled in the form for 2011, their feedback on the form resulted in several changes in the form for the following year. The registration form consisted of four areas: (1) personal characteristics of the client, (2) overview of the situation of the client prior to the start of counselling, (3) characteristics of the counselling process and (4) outcome of the counselling process (see Additional file 1). All forms returned by the counsellors were processed anonymously.

### Analysis

A description was given on frequencies of categories, focusing on characteristics of clients of counsellors working together with De Einder and the characteristics and outcome of the counselling process itself.

Based on the information provided by the clients, the counsellor classified the clients into four categories: (1) Terminal disease when cancer in a terminal or earlier phase or other disease with deadly diagnose was medically diagnosed, (2) Severe disease when a serious somatic disease (not terminal cancer) (e.g. heart failure, Chronic Obstructive Pulmonary Disease, Multiple Sclerosis/Amyotrophic Lateral Sclerosis, Cerebrovascular Accident) and/or serious psychiatric disease (e.g. severe depression) was medically diagnosed, (3) A non-severe disease (e.g. problems of old age, deterioration of mobility, problems of vision or hearing) or (4) No disease, when clients presented no physical or psychiatric complaints. For analysis purposes groups 1 and 2 and groups 3 and 4 were dichotomized into clients with a severe disease (including terminal diseases) and clients without a (severe) disease. Statistical significance was calculated by means of the Chi-square test. When requirements for the Chi-square Test were not met the Fisher’s Exact Test (two-sided) was used.

## Results

### Severity of the disease

A minority of the clients (5%) had a terminal disease, whereas 38% had a severe disease. Almost half of the clients (47%) had no (severe) disease. Of the remaining 10% of the clients the severity of or presence of a disease was unknown and they were excluded from the comparison between clients with a severe disease (including terminal disease) (n = 255) and clients without a (severe) disease (n = 280). For the latter group, in 2012 a distinction was made, showing that 54% had ‘no severe disease’ and 46% had ‘no disease’ (not in Table; see Additional files 2 and 3).

### Client characteristics

Almost two-thirds (61%) of the clients of counsellors working with De Einder were female (Table 2). More than half (56%) of the clients were 65 years or older. This group was larger for clients without a (severe) disease (70%) than for clients with a severe disease (44%). Eleven per cent of the clients were under 40 years old.

**Table 2:** Characteristics of people with face-to-face contact with a counsellor working with foundation De Einder^*

		Total ^A^(n = 595)	Severe disease (n = 255)	No (severe) disease (n = 280)	
		%	%	%	***P***-value ^B^
**Gender**					0.205
	Male	39	36	41	
	Female	61	64	59	
**Age**		^**X**^	^**X**^		**<0.001**
	18-39	11	18	5	
	40-64	33	39	25	
	65-79	29	24	37	
	≥80	27	20	33	
**Clients’ wish to end life at first contact and urgency** ^**C**^		^**Y**^	^**Y**^	^**Y**^	**<0.001**
	Wants to end life within 3 months	16	25	9	
	Wants to end life between 3 and 12 months	24	37	17	
	Wants to end life longer than 12 months away	23	13	25	
	No wish to end life	38	26	49	
**Former request for PAD**		^**Y**^	^**X**^	^**X**^	**<0.001**
	With no former request for PAD	61	49	76	
	With a former request for PAD	39	51	24	
**Outcome if requested for PAD** ^**D**^					0.825
	Refused	63	63	66	
	Pending	29	28	28	
	Granted	8	9	6	

^= Percentages are rounded therefore the total does not always add up to 100% exactly.

*= Missing observations between 0 and 37; missing is equal to or less than 5% (X) except for Y < 10.0%.

^A^= Including 60 clients with unknown severity of/or disease.

^B^= Pearson Chi-square test asymptotic significance 2-sided; p-value in bold = significant at a level of p=0.001.

^C^= This variable only for 2012. The N of the respective colums are N = 310 (for Total, including 25 clients with unknown severity of/or illness), N = 119 (for Severe disease) and N = 166 (for No (severe) disease).

^D^ **=** Only if a request for PAD. The N of the respective colums are N=218 (for Total, including 29 clients with unknown severity of/or illness), N = 125 (for Severe disease) and N = 64 (for No (severe) disease).

Over one third (38%) of the clients had no wish to end life. This group was larger for clients without a (severe) disease (49%) than for clients with a severe disease (26%). Of all clients, 16% wanted to end their life within three months and another 24% between three to twelve months. The group wanting to end life within a year was smaller for clients without a (severe) disease (26%) than for clients with a severe disease (62%).

The majority of all clients (61%) had not requested physician assistance in dying prior to or during counselling, often as a result of wishing to stay autonomous or judging they would not qualify for PAD (not in Table; See Additional file 4). Clients without a (severe) disease had requested physician assistance in dying less frequently (24%) than clients with a severe disease (51%).

Of the clients that requested physician assistance in dying, almost two thirds (63%) were confronted with a refusal, mostly due to not meeting the legal criteria of due care and/or moral objections of the physician (not in Table; See Additional file 5). For 29% the request was still pending and for 8% the request was granted.

### Sources of underlying suffering

Physical suffering was the most common mentioned reason of underlying suffering for contacting a counsellor and/or for having a wish to end life (42%), while psychiatric and psychological suffering accounted for respectively 23% and 16%. Almost one fifth (19%) mentioned no suffering at present (Table 3).

**Table 3:** Sources of underlying suffering of clients with face-to-face contact with a counsellor working with foundation De Einder (according to the counsellor)^*

		Total ^A^(n = 595)	Severe disease (n = 255)	No (severe) disease (n = 280)	
		%	%	%	***P***-value ^B^
**Main source of underlying suffering**		^X^	^X^	^X^	**<0.001**
	Physical suffering	42	55	30	
	Psychiatric suffering	23	35	8	
	Psychological suffering	16	7	26	
	No suffering at presence	19	4	36	
**Clarification of suffering** ^**C**^		^Y^	^X^	^Y^	
	*Physical suffering*				
	Problems of old age	17	9	27	**<0.001**
	Cancer	7	14	1	**<0.001**
	Dementia	6	11	1	**<0.001**
	Heart problems	4	4	4	0.788
	Rheumatism	3	5	1	0.017
	Lung problems	3	5	0	0.002
	Other physical problems^D^	19	28	9	**<0.001**
	*Psychiatric suffering*				
	Depression	16	19	11	0.007
	Personality disorder	13	17	7	**<0.001**
	Fear Disorders	5	6	4	0.140
	Other Psychiatric problems^E^	6	7	2	0.017
	*Psychological suffering*				
	Existential suffering (including weary of life/completed life)	19	10	29	**<0.001**
	Youth trauma (including child abuse)	6	6	5	0.412
	Loneliness	5	3	7	0.018
	Tiredness	3	4	3	0.320
	Other psychological problems^F^	5	3	7	0.040
	*No suffering at presence*				
	Selfdetermination/Be prepared	9	2	16	**<0.001**
	Avoiding dependency	5	1	10	**<0.001**
	No diseases or complaints	5	0	10	**<0.001**

^= Percentages are rounded therefore the total does not always add up to 100% exactly.

*= Missing observations between 1 and 53; missing is equal to or less than 5.0% (X) except for Y < 10.0%.

^A^= Including 60 clients with unknown severity of/or disease.

^B^= Pearson Chi-square test asymptotic significance 2-sided; p-value in bold means significant at a level of p=0.001.

^C^= Categories add up to more than 100% because more than one clarification per client possible.

^D^= Consisting of (amongst others): visual problems, cerebrovascular accident, osteoporosis/ortrosis, pain, multiple sclerosis, muscular disease.

^E^= Consisting of (amongst others): post traumatic stress syndrome, autism, eating disorder, attention deficit hyperactivity disorder.

^F^= Consisting of (amongst others): mourning, age, trauma, addiction, not wanting to suffer, financial problems.

Clients without a (severe) disease more often had no underlying source of suffering at present (36%) followed by physical suffering (30%) and psychological suffering (26%). The most often mentioned clarifications for the (absence of) underlying suffering for this group were existential suffering (including being weary of life) (29%), problems of old age (27%), wanting to be prepared for self-determination (16%), depression (11%), avoiding dependency (10%) of having no diseases or complaints (10%).

Clients with a severe disease most often mentioned physical suffering (55%) and psychiatric suffering (35%). The most often mentioned clarifications were other physical problems (28%), depression (19%), personality disorders (17%), cancer (14%), dementia (11%) and existential suffering (including being weary of life) (10%).

### Characteristics of counselling

Almost all clients (91%) started the counselling in the year of or one year prior to the year of registration. The majority of the clients (73%) had one face-to-face contact and in most cases (93%) the face-to-face contacts were complemented by other contacts by phone, email or in writing. More than half of the clients (54%) had 4 or more contacts (Table 4).

**Table 4:** Characteristics of counselling in cooperation with foundation De Einder^*

		Total ^A^(n = 595)	Severe disease (n = 255)	No (severe) disease (n = 280)	
		%	%	%	***P***-value ^B^
**Year of first contact**					0.512
	Year of data collection	78	81	77	
	One year before year of data collection	13	12	13	
	Two to eight years before year of data collection	9	7	10	
**Number of face-to-face consults**					0.055
	1	73	70	76	
	2	15	15	15	
	3 or more (3 to 21)	12	15	9	
**Number of total contacts** ^**C**^					0.109
	1	7	5	8	
	2-3	39	37	43	
	4-6	30	30	30	
	7 or more (7 to 37)	24	28	20	
**Involvement of other(s) in counselling and/or openness about counselling towards other(s)** ^**D**^					**0.001**
	Involvement and openness	25	34	20	
	Involvement but no openness	13	16	8	
	Involvement, openness unknown	11	11	10	
	No involvement but openness	15	17	14	
	No involvement nor openness	28	18	38	
	No involvement, openness unknown	10	4	10	
**Involvement of which other(s)** ^**E**^		^**X**^			
	Partner	41	40	44	0.625
	Children	35	39	30	0.288
	Friend	17	17	16	0.901
	Parents	7	10	2	*0.067*
	Brother/Sister	9	7	10	0.585
	Other family (cousin, grandchild)	5	6	5	*1.000*
	Medical Professional	1	1	0	*1.000*
	Non Medical Professional	1	0	2	*0.467*
**Counselling of explicit practical preparation (of gathering means and/or effectuation of suicide)**		^**X**^	^**X**^	^**X**^	**<0.001**
	Explicit practical preparation mentioned by counsellor	55	45	62	
	No explicit practical preparation mentioned by counsellor	45	55	38	

^= Percentages are rounded therefore the total does not always add up to 100% exactly.

*= Missing observations between 0 and 11; missing is equal to or less than 5.0% (X).

^A^= Including 60 clients with unknown severity of/or disease.

^B^= Pearson Chi-square test asymptotic significance 2-sided, unless *in cursive* Fisher’s Exact Test 2-sided; p-value in bold means significant at a level of p=0.001.

^C^= Consisting of face-to-face contacts, contacts in writing or by email, and/or contacts by phone.

^D^= This variable only for 2012. The N of the respective columns are N = 310 (for Total, including 25 clients with unknown severity of/or disease), N = 119 (for Severe disease) and N = 166 (for No (severe) disease).

^E^= This variable only for 2012 and if others involved; adds up to more than 100% because more than one answer possible. The N of the respective columns are N= 148 (for Total, including 13 clients with unknown severity of/or disease), N = 72 (for Severe disease) and N = 63 (for No (severe) disease).

In half of the cases (49%) the client involved another person whom the counsellor had spoken with or seen. Clients with a severe disease involved others more (61%) than clients without a (severe) disease (38%). Most often the involved others were a partner (41%) and/or children (35%). Of those who did not involve others in the counselling, more than half also had not told anyone about the counselling, reflecting 28% of all clients. Reasons for not involving others in the counselling were that it was regarded as a private matter, the client had a fear of the reactions of others, or the client was alone or had no network (Not in Table; See Additional file 6).

Finally, the counsellor discussed explicit practical aspects of non-PAS (concerning gathering the means for and the effectuation of the suicide) with over half of the clients (55%). This percentage was larger when the client had no (severe) disease (62%) than when the client had a severe disease (45%).

### Outcome of the counselling process

Almost one fifth (18%) of the clients were confirmed to have died at the moment the registration forms were filled out (Table 5). A few passed away through a natural death (2%) or received PAD (3%). The remaining 13% ended their life through non-PAS, the majority through taking lethal medication (90%) and a few through voluntarily refusing food and fluid (5%) or oxygen deprivation by inhalation of inert gas (5%) (not in Table; See Additional file 7). Clients with a severe disease more often passed away within the registered period (24%) than clients who had no (severe) disease (11%).

**Table 5:** Characteristics of outcome of counselling in cooperation with foundation De Einder^*

		Total ^A^ (n = 595)	Severe disease (n=255)	No (severe) disease (n=280)	
		%	%	%	***P***-value ^B^
		^X^	^X^	^X^	
**Counselling ended due to passing away of client**		**18**	**24**	**11**	**<0.001**
	*Natural death*	*2*	*3*	*1*	
	*Physician Assisted Dying*	*3*	*4*	*1*	
	*Non Physician Assisted Suicide*	*13*	*17*	*9*	
**Counselling ended for other reasons**		**7**	**7**	**7**	
	*Client wants to continue living*	*2*	*2*	*1*	
	*Referred to physician/treatment*	*1*	*2*	*1*	
	*Ended after preparing method*	*4*	*3*	*4*	
**Counselling on hold** *(method non-PAS prepared; pending future contacts)*		**22**	**17**	**29**	
**Counselling on-going**		**54**	**52**	**54**	
	*General information & support*	*17*	*18*	*19*	
	*Mental & Practical counselling*	*37*	*34*	*35*	

^= Percentages are rounded therefore the total does not always add up to 100% exactly.

*= Missing observations between 5 and 20; missing is equal to or less than 5.0% (X).

^A^= Including 60 clients with unknown severity of/or disease.

^B^= Pearson Chi-square test asymptotic significance 2-sided. Result for main categories (in bold); p-value in bold means significant at a level of p=0.001.

In another 7% the counselling ended due to other reasons (e.g. having a wish to live on, referred for treatment elsewhere, or because the client had prepared their non-PAS). Theoretically this group of clients could have died without the counsellor knowing and therefore the number of deceased clients could be underestimated.

Another 22% of the clients also prepared their method to end their life but the counselling was regarded to be ‘on hold’, pending future contacts so the client could check on their medication or discuss precautions for performing non-PAS. Clients ‘on hold’ were more common for those without a (severe) disease (29%) than for those with a severe disease (17%).

For the remaining clients (54%) the counselling was on-going at the moment of registration. For 17% the counselling (still) consisted of offering general information, a listening ear and/or moral support, while in 37% the client was being counselled for the mental aspects of the wish to end life and/or practical preparation for non-PAS.

## Discussion and conclusions

### Summary of results

More than half of the clients of counsellors working with foundation De Einder are over 65 years old. More than one third of the clients have no wish to end life and almost two thirds of the clients have not requested physician assistance in dying. Sixteen per cent of all clients wish to end life within three months.

In half of the cases others are involved in the counselling, often the partner and/or children. More than half of the clients receive explicit practical information on non-PAS, while only 13% of all clients have ended life through non-PAS – most often through an overdose of lethal medication.

There are differences in characteristics of clients without a (severe) disease and clients with a severe disease. The clients without a (severe) disease are older, more often have no wish to end life, request physician assistance in dying less often, have more problems of old age and existential suffering and more often want to be prepared for self-determination. Less often they have other persons involved in the counselling, more often receive explicit practical information and less often pass away within the registered period.

### Strengths and shortcomings

The 100%-response rate of counsellors Foundation De Einder refers to, gives a reliable view on the client group. While in recent years more attention has been given to the existence of non-PAS [5, 9–14, 19–21], research into the assistance offered by non-physicians was unavailable. This research is the first to provide insight into counselling for non-PAS in a quantitative way and has been able to include a large group of clients (N = 595).

However, results cannot be generalized to non-PAS in general because deceased clients of counsellors working together with foundation De Einder only form a small group of all people that died through non-PAS. Secondly, other assisting non-physicians, like volunteers from other right-to-die organisations or relatives and friends, may have a different position and approach towards non-PAS than professional counsellors. Furthermore, information bias may have influenced the data. Information about the clients is collected through counsellors and the available information is dependent on what clients share with the counsellor.

### Many clients do not have a death wish

Over one third (38%) of clients of counsellors working with De Einder have no wish to end life, while 16% of the clients have a wish to end life within three months. This raises the question what reasons these different groups of clients have for receiving counselling for non-PAS.

### Looking for a peaceful death

The first reason for receiving counselling for non-PAS may be that especially clients with a severe disease are looking for a peaceful death for current suffering. Almost two thirds of these clients wish to end life within a year. Half of the clients with a severe disease have requested physician assistance in dying, which resulted in a denial in two thirds of the requests. A quarter of the clients with a severe disease have passed away, of which two thirds died by non-PAS.

It is plausible that some of these clients may have had difficulties receiving PAD. Research has shown that physicians are reluctant to offer PAD to patients with psychiatric problems or dementia [5]. These disease are reported by counsellors to be more common with clients with a severe disease. Also moral objections of the physician have played a role in the denial of the requests. Finally, clients who believe they do not qualify for PAD and wish to stay autonomous may lead them to seek counselling rather than ask for PAD.

Since the opening of the End of Life Clinic in The Hague, the Netherlands – consisting of ambulatory teams that help people with a death wish, if they fall within the scope of the Dutch PAD law – patients have another possibility instead of asking their own physician. However, there will always be people falling outside the scope of the Dutch PAD law or who wish to stay autonomous. A foundation like De Einder provides in the possibility for these people to carefully deliberate on their wish to end life and prepare for non-PAS.

### Looking for reassurance

A second reason for receiving counselling for non-PAS may be that people are looking for reassurance in anticipation of prospective suffering. These clients seem to be more clearly distinguished in the group of clients without a (severe) disease. Half of these clients have no wish to end life and a considerable number of these clients want to be prepared for self-determination and/or avoid dependence on others. While almost two thirds of the clients have been explicitly informed, for example on gathering the means for non-PAS, only 9% has ended life through non-PAS and almost one third put the counselling ‘on hold’ after having prepared a method of non-PAS.

The idea that people are looking for reassurance to prevent future suffering is probably reflected in the large amount of patients requesting physician assistance in dying *for in due time* (about 33,900 in 2010) as compared to the patients explicitly requesting physician assistance in dying *for current situations* (about 13,400 in 2010) [5]. This reassurance to prevent future suffering can also explain why only a minority of patients, that are deemed eligible to receive assistance with dying from the Swiss right-to-die organisation Dignitas, actually make use of this assistance. They seem to regard this possibility as an ‘emergency exit’ option for when the deterioration of their health may become unbearable [22, 23]. This idea of reassurance by having an emergency exit option available, has also been reported in interviews with elderly people who are weary of life [19]. The wish for reassurance can be related to the idea that death wishes serve as “a way of autonomous protection against the threat of continued living, feeling and thinking”. [24]. The counselling and having the knowledge to be able to prepare or being prepared for non-PAS may give feelings of reassurance and the perception of control for these clients.

### Implications

In recent years, non-PAS through voluntarily refusing food and fluid or taking lethal medication has gotten more attention in the Netherlands [5, 9–14, 19–21]. About half of the Dutch general public finds it acceptable if a professional assists by informing on non-PAS [20]. The Royal Dutch Medical Association has explicated the role of the physician concerning non-PAS. When the patient decides to voluntarily refuse food and fluid, then the physician must have due regard for the care provided by a good care provider [21, 25]. When the patient opts for taking lethal medication, then the physician can hold conversations about the topic and provide information. The physician can, but is not obligated to, refer the patient to available resources and experts [21]. As the data has shown, counsellors working together with Foundation De Einder have experience with people ending their lives through non-PAS with lethal medication. Therefore they could be a valuable source of information and knowledge and we recommend that physicians also consult them.

If non-PAS is to be distinguished from ‘mutilating’ suicide, then another approach than suicide prevention or crisis intervention is asked for by health care professionals. Berghmans et al. notices that “policy for the past years has mainly focussed on suicide prevention, as an act of justified paternalism that it is better (and morally obligatory) to save life than to respect the wish of the person. However, from an ethical point of view, it can be argued that preventing rational suicides by limiting the freedom and liberty of a competent person cannot be justified on paternalistic grounds” [26]. In this line of thinking, we recommend to complement suicide-prevention with ‘suicide-attempt prevention’, a term coined by Minelli from the Swiss organisation Dignitas. Hereby people with death wishes can talk openly about the wish to die and where possible a sensible and attainable solution to their unbearable situation can be searched for. When this is not possible also non-PAS can be discussed. Minelli suggests this approach might be able to prevent lonely suicide attempts [27]. While this is partly due to open communication about the death wish – a feature also shared with many suicide prevention organisations – another reason is the relief experienced by offering the possibility to an accompanied suicide by Dignitas. This approach seems to be in line with the work offered by counsellors working together with foundation De Einder. Respect for the autonomy of the person, the acceptance of the possibility of suicide and the provision of information on non-PAS are key features.

Evidence of the suggested effect that suicide attempt prevention prevents lonely suicide attempts cannot, however, be deducted from the available data. We recommend follow-up research into the results of the counselling, and interviewing clients and counsellors and others involved, to help answer these questions. The approach of suicide-attempt-prevention does, however, offer physicians a way to openly communicate about wishes to die with the patient. It is argued that discussing death wishes – even outside the context of PAD – are important because if people feel unable to talk about them, their quality of life may be further diminished [28]. The Royal Dutch Medical Association recommends having a conversation on the subject of life’s end and death wishes as a way to get to know the patient better [29]. Actually, we also recommend discussing non-PAS so to offer a chance to give the patient an improved perception of control, hopefully leading to a better level of coping and more quality of life.

### Approval by Ethics Committee

This research has been granted an exemption from requiring ethics approval because the research does not require approval under the Dutch law on Medical-Scientific Research with Humans (Wet Medisch-Wetenschappelijk Onderzoek met Mensen; WMO). This approval has been granted by the VU Medical Center Medical Ethics Committee.

### Consent

All data has been received anonymously through the board of Foundation De Einder and the counsellors the foundation refers to.

## Authors’ information

MH: Psychologist, PhD student and junior researcher. HRWP: Sociologist, PhD and senior researcher end-of-life research. BOP: Health Scientist and professor end-of life research.

## Electronic supplementary material

Additional file 1: **Appendix registration form 2012.** (PDF 62 KB)

Additional file 2: **Severity of illness.** (PDF 26 KB)

Additional file 3: **Distinction between no disease and no severe disease.** (PDF 34 KB)

Additional file 4: **Reasons for not requesting PAD.** (PDF 29 KB)

Additional file 5: **Reasons for denial of request for PAD.** (PDF 27 KB)

Additional file 6: **Reasons not involving others in counselling.** (PDF 27 KB)

Additional file 7: **Method Non-Physician Assisted Suicide.** (PDF 27 KB)

## Abbreviations

Non-PAS: Non-physician assisted suicide
PAD: Physician assisted dying (entailing Termination of life by the physician on request of the patient and Physician-assisted suicide).
