# Supplementary material for: Cross-sectional research into counselling for non-physician assisted suicide: who asks for it and what happens?
Source: BMC Health Serv Res. 2014 Oct 2;14:455. doi: 10.1186/1472-6963-14-455 (PMC4283078; doi:10.1186/1472-6963-14-455)
Supplement: Supplementary file 1 — Additional file 1: Appendix registration form 2012. (PDF 62 KB) [file 12913_2014_3541_MOESM1_ESM.pdf]

Additional File 1: Appendix registration form 2012

Item	Selected option
1. Personal characteristics of the client	
Postal Code	4 digits of Dutch postal code<br>Name of country, if abroad<br>Unknown
Gender	Male<br>Female<br>Unknown
Year of birth	4 digits<br>Unknown
2. Overview of the situation of the client prior to start of counselling	
Main motivation to request counselling	Physical suffering<br>Psychiatric suffering<br>Psychological suffering<br>No suffering at present<br>Unknown
Current disease(s), diagnosis or situation	Open-ended questions. More than one answer possible.<br>Several examples preselected:<br>Physical: E.g. cancer, problems of old age, dementia, heart problems.<br>Psychiatric: E.g. depression, personality disorders, fear disorder<br>Psychological: E.g. existential suffering, loneliness, youth trauma.<br>No current suffering: E.g. wanting autonomy, completed life.<br>Unknown
Severity of disease	Terminal disease<br>Severe disease<br>No severe disease<br>No disease/not applicable<br>Unknown
Presence and urgency of death wish at start of counselling	No death wish present<br>Death wish present, wants to end life within three months<br>Death wish present, wants to end life between 3 to 12 months<br>Death wish present, wants to end life more than one year away<br>Unknown
Request for assistance in dying to a physician	No request for PAD<br>Request for PAD denied<br>Request for PAD pending<br>Request for PAD granted<br>Unknown
Explication on request	Open-ended question. More than one answer possible.<br>Several examples preselected:<br>No request: E.g. staying autonomous, not wanting to burden the physician.<br>Request denied: E.g. not meeting legal criteria of due care.<br>Request pending: E.g. physician has to think about it<br>Request granted: E.g. granted for near future, granted for in due time.<br>Unknown<br>Not applicable
3. Characteristics of the counselling process (including client system)	
Number of face-to-face contacts in 2012	Any number<br>Unknown
Number of other contacts in 2012	Any number<br>Unknown

Total number of contacts in 2012	Calculated automatically from two prior columns
Month of first contact	Jan – Dec<br>Unknown
Year of first contact	1995 – 2012<br>Unknown
Number of face-to-face contacts since start of counselling	Any number<br>Unknown
Number of other contacts since start of counselling	Any number<br>Unknown
Total number of contacts since start of counselling	Calculated automatically from two prior columns
Character of counselling	Open-ended question. More than one answer possible.<br>Several examples preselected:<br>Referral: E.g. referral to physician, suicide prevention, End of Life Clinic<br>General information: E.g. about goals foundation, about euthanasia law.<br>Mental counselling: E.g. motivation, emotional, role of others.<br>Practical counselling: E.g. explicit information on methods, preparing,<br>Counselling ended: E.g. deceased, treatment elsewhere, continue living
Involvement of others during counselling	Yes<br>No
Reason for no others involved	Open-ended question.<br>Several examples preselected:<br>No social network/Alone<br>Fear of reactions<br>Regarded as a private matter<br>Unknown<br>Not applicable
Relationship of others involved	Open-ended question. More than answer possible.<br>Several examples preselected:<br>E.g. partner, (grand)child(ren), parents, siblings, friends.<br>Not applicable
Number of others involved	Any number<br>Not applicable
Attitude of involved others	Positive/Supportive<br>Negative/Critical<br>Mixed/Ambiguous<br>Unknown<br>Not applicable
Openness about counselling towards others	Yes<br>No<br>Unknown
Reason for no openness towards others	Open-ended question.<br>Several examples preselected:<br>No social network/Alone<br>Fear of reactions<br>Regarded as a private matter<br>Unknown<br>Not applicable
Relationship of other towards whom clients is open	Open-ended question. More than answer possible.<br>Several examples preselected:<br>E.g. partner, (grand)child(ren), parents, siblings, friends.<br>Not applicable
Number of other towards whom client is open	Any number<br>Unknown<br>Not applicable
Attitude of others	Positive/Supportive

towards whom client is open	Negative/Critical<br>Mixed/Ambiguous<br>Unknown<br>Not applicable
4. Outcome of the counselling process	
See also character of counselling at 3.	See also character of counselling at 3.
Manner of death, if client deceased.	Not deceased<br>Unknown if deceased<br>Natural death<br>Physician-assisted dying<br>Lethal medication<br>Voluntary refusing food and fluid<br>Helium<br>Dignitas<br>Hanging/Choking<br>Jumping train<br>Jumping height<br>Drowning<br>Unknown manner of death
When deceased: month	Jan – Dec<br>Unknown<br>Not applicable
When deceased: year	2011<br>2012<br>2013<br>Not applicable
Follow up counselling with bereaved ones	Yes<br>No
Other information on follow up counselling	Open- ended question.
5. Other	
Additional remarks about whatever deemed relevant	Open-ended question
