# Supplementary material for: Cross-sectional research into counselling for non-physician assisted suicide: who asks for it and what happens?
Source: BMC Health Serv Res. 2014 Oct 2;14:455. doi: 10.1186/1472-6963-14-455 (PMC4283078; doi:10.1186/1472-6963-14-455)
Supplement: Supplementary file 2 — Additional file 2: Severity of illness. (PDF 26 KB) [file 12913_2014_3541_MOESM2_ESM.pdf]

Additional File 2: Severity of illness
(N = 595)

	Frequency	Percentage
Terminal disease	30	5
Severe disease	225	38
No (severe) disease	280	47
Unknown	60	10
Total N	595	100
