# Supplementary material for: Cross-sectional research into counselling for non-physician assisted suicide: who asks for it and what happens?
Source: BMC Health Serv Res. 2014 Oct 2;14:455. doi: 10.1186/1472-6963-14-455 (PMC4283078; doi:10.1186/1472-6963-14-455)
Supplement: Supplementary file 3 — Additional file 3: Distinction between no disease and no severe disease. (PDF 34 KB) [file 12913_2014_3541_MOESM3_ESM.pdf]

Additional File 3: Distinction between no disease and no severe disease
(Only for data 2012, N = 310)

		Frequency		Percentage	
Terminal disease		12		4	
Severe disease		107		35	
No (severe) disease		166		54	
	No severe disease		*89*		*29*
	No disease		*77*		*25*
Unknown		25		8	
	Total N	310		100	

		Frequency		Percentage	
No (severe) disease		166		100	
	No severe disease		*89*		*54*
	No disease		*77*		*46*
