# Supplementary material for: Cross-sectional research into counselling for non-physician assisted suicide: who asks for it and what happens?
Source: BMC Health Serv Res. 2014 Oct 2;14:455. doi: 10.1186/1472-6963-14-455 (PMC4283078; doi:10.1186/1472-6963-14-455)
Supplement: Supplementary file 4 — Additional file 4: Reasons for not requesting PAD. (PDF 29 KB) [file 12913_2014_3541_MOESM4_ESM.pdf]

Additional File 4: Reasons for not requesting PAD
(only for data 2012 and if no request for PAD, N = 187)

	Frequency	Percentage
Client judges PAD not possible	41	22
Client wishes to stay autonomous	39	21
Other reasons	12	6
Clients doesn't want to burden physician	11	6
PAD not discussable with physician / moral objections	11	6
Client has fear of being sectioned	7	4
Unknown / Missing	66	35
Total N	187	100
