# Supplementary material for: Cross-sectional research into counselling for non-physician assisted suicide: who asks for it and what happens?
Source: BMC Health Serv Res. 2014 Oct 2;14:455. doi: 10.1186/1472-6963-14-455 (PMC4283078; doi:10.1186/1472-6963-14-455)
Supplement: Supplementary file 5 — Additional file 5: Reasons for denial of request for PAD. (PDF 27 KB) [file 12913_2014_3541_MOESM5_ESM.pdf]

Additional File 5: Reasons for denial of request for PAD
(Only for data 2012, and if request for PAD denied, N = 71)

	Frequency	Percentage
Patient doesn't meet criteria of due care	46	65
Moral objections	9	13
Other reasons	12	17
Unknown / Missing	4	6
Total N	71	100
