# Supplementary material for: Cross-sectional research into counselling for non-physician assisted suicide: who asks for it and what happens?
Source: BMC Health Serv Res. 2014 Oct 2;14:455. doi: 10.1186/1472-6963-14-455 (PMC4283078; doi:10.1186/1472-6963-14-455)
Supplement: Supplementary file 6 — Additional file 6: Reasons not involving others in counselling. (PDF 27 KB) [file 12913_2014_3541_MOESM6_ESM.pdf]

Additional File 6: Reasons for not involving others in counselling
(Only for data 2012 and if others not involved, N = 162)

	Frequency	Percentage
Regarded as a private matter	55	34
Fear of reactions of other	43	27
No social network or alone	41	25
Other reasons	8	5
Unknown	15	9
Total N	162	100
