# Supplementary material for: Cross-sectional research into counselling for non-physician assisted suicide: who asks for it and what happens?
Source: BMC Health Serv Res. 2014 Oct 2;14:455. doi: 10.1186/1472-6963-14-455 (PMC4283078; doi:10.1186/1472-6963-14-455)
Supplement: Supplementary file 7 — Additional file 7: Method Non-Physician Assisted Suicide. (PDF 27 KB) [file 12913_2014_3541_MOESM7_ESM.pdf]

Additional File 7: Methods of Non-Physician Assisted Suicide
(Only if passed away through Non-PAS, N = 76)

	Frequency	Percentage
Lethal medication	68	89
Voluntary refusal of food and fluid	4	5
Oxygen deprivation by inhalation of inert gas	4	5
Total N	76	100
